# Antidepressant activity of an aqueous extract from okra seeds†

**DOI:** 10.1039/c8ra03201g

**Published:** 2018-09-21

**Authors:** Fangbo Xia, Chenchen Li, Mengqiu Li, Yonghong Liao, Xinmin Liu, Jianyong Si, Qi Chang, Ruile Pan

**Affiliations:** Institute of Medicinal Plant Development, Peking Union Medical College, Chinese Academy of Medical Sciences No. 151, North Road Malianwa, Haidian District Beijing 100193 PR China rlpan@implad.ac.cn

## Abstract

Faced with the increasing incidence of major depression disorder (MDD) and the unsatisfactory effect of current drugs, there has been growing attention on the relation between dietary supplements and MDD prevention. In this research, the antidepressant activity of okra seed extract (OSE) was evaluated with behavioral tests including an open field test, tail suspension test (TST), forced-swimming test (FST) and novelty suppressed feeding test (NSFT) for sub-chronic treatment and chronic sleep-interruption (CSI) animal models. The chemical constituents of OSE were identified by using UPLC-DAD/Q-TOF MS. To investigate the mechanism, the prefrontal cortex and hippocampus were collected to determine neurotransmitters, total antioxidant capacity (T-AOC), superoxide dismutase (SOD) and malondialdehyde (MDA). Blood serum was prepared for the determination of corticosterone (CORT) and adrenocorticotropic hormone (ACTH). Results demonstrated that OSE possessed an antidepressant effect in both sub-chronic treatment and CSI animal models through suppressing the hyperactivation of the hypothalamic–pituitary–adrenal (HPA) axis, alleviating oxidative stress and regulating neurotransmitter levels in the hippocampus and frontal cortex. Besides, chemical analysis based on the UPLC-DAD/ESI-Q-TOF MS approach showed that OSE mainly contained catechin and quercetin derivatives. The present study provided a scientific basis for developing okra seeds to be a dietary supplement for MDD prevention.

## Abstract 2

Faced with the increasing incidence of major depression disorder (MDD) and the unsatisfactory effect of current drugs, there has been growing attention on the relation between dietary supplements and MDD prevention.

## Graphical abstract

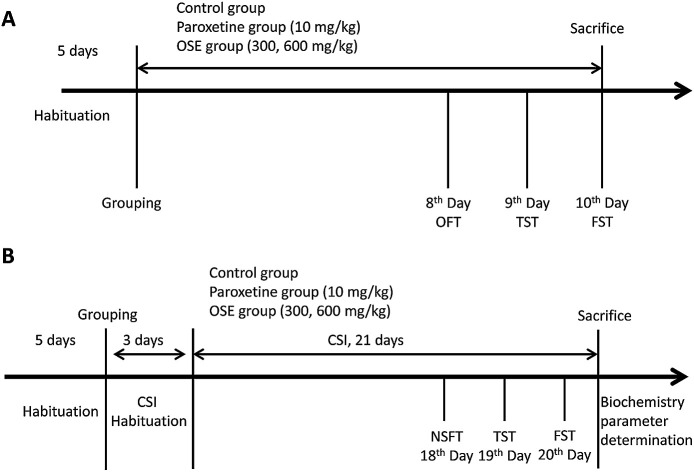

## 1. Introduction

Major depression disorder (MDD) is a common brain disorder, which has been considered a leading cause of disease burden worldwide.^1^ MDD is not only characterized by profound dysregulation of emotion or mood, interest loss and low self-worth, but also associated with cognitive dysfunction, disturbed sleep and appetite, fatigue, and other endocrine or metabolic alterations.^2^ Due to the poor understanding of the etiology, the available antidepressants in clinical practice still focus on the monoamine disorder hypothesis based on the theory that the monoamine neuro-transmitters are depleted in major depression.

Most of current antidepressants are not completely effective (only 33% of depressed patients are sensitive to their first antidepressant medication).^3^ What's worse, they are associated with many serious adverse effects, such as cardiac toxicity, sexual dysfunction, body weight gain and sleep disorder.^4^ Recently, there has been growing attention on the relation between dietary supplement (and potential nutritional deficiencies) and mental health. It has been presented that diet and nutrition offer key modifiable targets for the prevention of mental disorders, which play a fundamental role in the promotion of mental health.^5^ From this point of view, it is important to find a safer, better-tolerated antidepressant from natural plants.

Plant polyphenols are widespread in the natural green plants, and are important secondary metabolites. Plant polyphenols involve various biological activities, such as antioxidant,^6^ neuroprotection,^7,8^ anti-inflammation^9,10^ and curing neurodegenerative and cardiovascular diseases.^11^ Recently, several phenolic phytochemicals, such as resveratrol,^12^ curcumin,^13^ tea polyphenols,^14^ ferulic acid,^15^ have been found to exhibit antidepressant-like effects by protecting nerve cells from oxidative damage, and resulting in the remission and functional recovery from oxidative damage.

Okra (*Abelmoschus esculentus* (L.) Moench) is one of the members of family *Malvaceae*. It is known by many names, such as lady's finger, bamyah and bhindi, and its pods are eaten raw as well as cooked as a vegetable. Okra is of African origin where it has been cultivated for more than 4000 years, now is also grown in different tropical and warm temperature regions of the world, like Greece, Iran, Egypt, India, Japan, southern United States and Turkey, Philippines and China. The previous studies has proved that okra pods contained high contents of polyphenols and flavonoids^16^ and possessed antioxidant, neuroprotective, antidiabetic, antihyperlipidemic effects.^17–20^ Besides, our previous study demonstrated that the contents of polyphenols in okra seeds is about 24 times as much as that in okra skin with Folin–Ciocalteu method, and also proved that the okra seed water extract (OSE) possessed the strong antioxidant and anti-fatigue effects.^21^ Thus, it is speculated that OSE may have the promising antidepressant function. As for the quality control of okra seeds, it has been proved that the contents of total polyphenols played important roles. To clarify the chemical composition of polyphenols, UPLC-DAD/Q-TOF MS approach was applied to study the chemical constituents of OSE in this research.

The present study aims to illuminate the antidepressant activity of OSE with sub-chronic and chronical sleep interruption (CSI) models. Besides, the phenolic compounds of OSE are identified with UPLC-DAD/Q-TOF MS.

## 2. Experimental

### 2.1. Plant material

Fresh okra pods, authenticated by Professor Ruile Pan (the Institute of Medicinal Plant, Chinese Academy of Medical Sciences and Peking Union Medical College, Beijing, China), were gathered from Zhengzhou, Henan Province, China in July, 2015 and the voucher specimens (no. 20150809) have been deposited in Herbarium of the institute.

### 2.2. Sample preparation

Five kilograms of fresh okra pods were divided into okra seeds (1 kg) and okra skins (4 kg). The okra seeds were extracted with 3000 mL boiling water for 3 times (each time for 1 h). Each filtered liquid was combined and concentrated under vacuum, to yield okra seed extract (OSE, 40.5 g). The content of total polyphenols in OSE was 29.5%, which was determined using our previous method.^21^ Sample was stored at −20 °C for subsequent chemical analysis and animal experiments.

### 2.3. Chemicals and reagents

Paroxetine (PAR) was purchased from the National institutes for Food and Drug Control (Beijing, China). The standard substances of dopamine (DA), 5-hydroxytryptamine (5-HT), norepinephrine (NE), epinephrine (E) and acetylcholine (Ach) were purchased from Sigma-Aldrich Co. (St. Louis, MO, USA). Corticosterone (CORT) and adrenocorticotrophic hormone (ACTH), total antioxidant capacity (T-AOC) assay kit (FRAP method), malondialdehyde (MDA) and superoxide dismutase (SOD) were purchased from Nanjing Jiancheng Biotechnology Institute (Nanjing, China). Acetonitrile and ammonium formate of HPLC grade were obtained from Thermo Fisher Scientific (MA, USA). Ultrapure water was obtained from a Milli-Q water purification system (Millipore, USA). All other chemicals used for analysis were analytical grade, obtained from Beijing Chemical Works (Beijing, China).

Fourteen reference compounds for UPLC-DAD/Q-TOF MS analysis were isolated from okra seeds by our research group. Their structures were identified by ^13^C NMR, ^1^H NMR and HR-MS, and the purities determined with HPLC method were all over 95%. Their structures were shown in ESI 1.†

### 2.4. UPLC-DAD/Q-TOF MS analysis

OSE (100 mg) was dissolved in chromatographic methanol (10 mL), and the solution was filtered through 0.22 μm polytetrafluoroethylene filters before analysis.

A Waters Acquity Ultra Performance LC system (Waters Corporation, Milford, MA, USA) equipped with a photo diode array detector (DAD, 190–400 nm) was used for analysis, and the system was controlled by the MassLynx V4.1 software (Waters Co., USA). Separations were performed using a Waters HSS T3 column (2.1 mm × 100 mm, 1.7 μm). The mobile phase was composed of methyl alcohol (A) and 0.1% formic acid–water (B). The following solvent gradient system: 3−10% A from 0 to 10 min, 10–65% A from 10 to 35 min, 90% A from 35 to 37 min. The flow rate was 0.3 mL min^−1^, and column temperature was set at 35 °C. The injection volume was 3 μL.

The mass spectrometric data were collected using a Q-TOF analyzer in a SYNAPT HDMS system (Waters Corporation, Milford, MA, USA) in negative ion modes. The optimization parameters were set as following: the source temperature was set at 100 °C with a cone gas flow of 100 L h^−1^, a desolvation gas temperature at 350 °C and a desolvation gas flow of 800 L h^−1^. For the negative ion modes, the capillary voltage was set to 2.5 kV, and the cone voltage was set to 40 V. Centroid data were collected from *m*/*z* 100 to 1500 with a scan time of 0.1 s and an interscan delay of 0.02 s over analysis time. Leucine-enkephalin was used as the lock mass (*m*/*z* 554.2615 in negative ion mode) at a concentration of 0.5 mg mL^−1^ with a flow rate of 80 mL min^−1^.

### 2.5. Animals

Male ICR mice (17–19 g, 8 weeks) were purchased from the Vital River Laboratories (Qualified no. SCXK 2012-0001, Beijing, China). The mice were housed in groups of 6 animals per cage under a constant temperature (23 ± 2 °C) and humidity (50% ± 10%) on a 12/12 h light/dark cycle (lights on from 08:00 to 20:00). Mice had free access to standard chow diet and sterilized drinking water in the SPF animal house. All experimental procedures were conducted under the supervision and approval of the Academy of Experimental Animal Center of the Institute of Medicinal Plant Development and in strict accordance with the NIH Guide for the Care and Use of Laboratory Animals (Eighth edition).

### 2.6. Experimental design

#### 2.6.1. Antidepressant effect of OSE on sub-chronic treatment mice

After five days of acclimatization, the mice were randomly divided into four groups (12/group): control, paroxetine group (10 mg kg^−1^ body weight), OSE treatment groups (300, 600 mg kg^−1^ body weight). Distilled water (control), paroxetine solution and OSE solutions were orally administered to the corresponding group for 10 days.

**Fig. 1:**
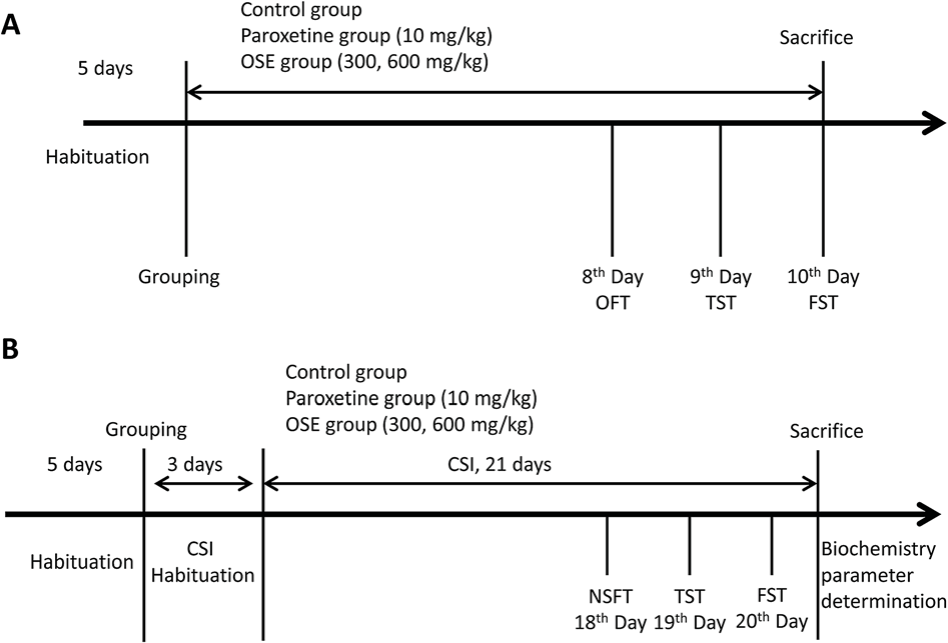
Experiment design for antidepressant effect of okra seed extract (OSE) on sub-chronic treatment mice (A); experimental design for antidepressant effect of OSE on chronical sleep interruption (CSI) mice (B). OFT: open field test; TST: tail suspension test; FST: forced swim test; NSFT: novelty suppressed feeding test.

Behavioral tests were carried as follows: open field test (8th day); tail suspension test (9th day) and forced-swimming test (10th day). After finishing the behavioral tests, the mice were sacrificed with ether anesthesia. Detail experimental scheme is displayed in Fig. 1(A).

#### 2.6.2. Antidepressant effect of OSE on chronical sleep interruption (CSI) mice

After five days of acclimatization, the mice were randomly divided into five groups (12/group): normal, control, paroxetine group (10 mg kg^−1^ body weight), OSE treatment groups (300, 600 mg kg^−1^ body weight). Distilled water (normal and control), paroxetine solution and OSE solutions were orally administered to the corresponding group for 21 days.

All groups except for the normal group were subjected to CSI using the Sleep Interruption Apparatus (SIA, a computer controlled rotating drum, Chinese no. 201210356645.X) from 8 am to 11 am each day. The parameters of SIA were set as follows: rotation speed, 60 s per revolution; rotation frequency, rotating 1 min after 2 min pause.

Mice in the control group were housed in a static SIA copy apparatus, and the other groups were taken back to the animal room for 3 days for acclimatization. After 3 days of CSI habituation, the 21 day CSI period began. The mice were provided with water and food in the SIA during the CSI acclimatization period, the 21 day CSI period and the behavioral test periods. Behavioral tests were carried as follows: novelty suppressed feeding test (18th day), tail suspension test (19th day) and forced-swimming test (20th day). After finishing the behavioral tests, the mice were sacrificed for biochemistry parameter analysis. Detail experimental scheme is displayed in Fig. 1(B).

### 2.7. Behavioral tests

#### 2.7.1. Open field test (OFT)

Locomotor activity was measured using the open field test following our previous method.^21^ Briefly, the mice were evaluated automatically using an open field computer-aided controlling system developed by Institute of Medicinal Plants, Chinese Academy of Medical Sciences. The apparatus consists of four metal tanks (30 cm in diameter, 40 cm in height) with a video camera fixed at the top. Experiments were performed in a quiet room, and the apparatus was illuminated by a light source of 120 lux on the ceiling. One hour after dosing, each mouse was placed at the center of the metal tank and allowed to explore freely for 2 min and the distance travelled in 4 min, which is an index of locomotor activity, was calculated using software. Four mice were tested simultaneously.

#### 2.7.2. Novelty suppressed feeding test (NSFT)

The NSFT was performed based on previous publication.^22^ Firstly, mice were fasted for 24 hours, and then a single food pellet (regular chow) was placed in the center of wood box (50 cm × 50 cm × 20 cm). The animal was placed in the box from any direction, and observed by the image pickup system for 5 minutes. The latency time to bite for the mice were recorded from being put in the box to the mice picking up and eating the food. The activity level of the mice and the length of eating time reflects the anxiety/depression degree. Lastly, the mice were returned in its cage and the amount of food consumption in 5 minutes were recorded in order to exclude the influence of the appetites.

#### 2.7.3. Tail suspension test (TST)

The TST was performed according to previous publication with minor modification.^23^ In short, mice were suspended 50 cm above the floor by adhesive tape placed approximately 1 cm from the tip of the tail. The test was videotaped, and immobility time was measured for 6 min. Immobility was defined as the absence of any limb or body movements. The mice hang passively and completely motionless with the exception of respiration effect. During the test, the mice were separated from each other to prevent visual and acoustic associations. The number of seconds spent immobile was recorded. Observers were blind to the group treatment of the mice.

#### 2.7.4. Forced swim test (FST)

The forced swim test was conducted following our previous literatures.^24^ Mice were placed in a 20 cm diameter × 35 cm height plastic cylinder filled with 20 cm height water (23–25 °C). The test was videotaped, and immobility time was measured. The definition of immobility was the absence of all movements with the exception of motions required to maintain the animal's head above the water. The results are expressed as the time spent immobile during the last 4 min of the 6 min session. Observers were blind to the group treatment of the mice.

### 2.8. Tissue collection and biochemical parameter measurement

After the forced swim test, mice were sacrificed under ether anesthesia. The prefrontal cortexes and hippocampals were collected, and immediately put into liquid nitrogen, and then stored at −80 °C until the determination of neurotransmitter, T-AOC, SOD and MDA. Blood serum was prepared for the determination of CORT and ACTH.

#### 2.8.1. Analysis of neurotransmitters in the prefrontal cortex and hippocampal

The concentration of six neurotransmitters, including dopamine (DA), noradrenaline (NE), 5-hydroxytryptamine (5-HT), acetylcholine (Ach) and epinephrine (E) in the prefrontal cortex and hippocampal of mice were simultaneously determined using dihydroxy-benzoic acid (DHBA) as an internal standard by a previously reported method with slight modification.^25^ Briefly, the prefrontal cortex and hippocampus of mice were homogenized in 100 μL of a ice-cold 0.2% formic acid aqueous solution. Then 100 μL of the homogenate was mixed with 200 μL of ice-cold acetonitrile containing 0.2% formic acid, and then centrifuged at 12 000 rpm for 10 min at 4 °C. An aliquot of supernatant (100 μL) was added to 10 μL of internal standard solution (300 μg mL^−1^, 3,4-dihydroxybenzylamine, DHBA), 50 μL of the prepared sample was injected into a LC-MS/MS instrument for assay.

LC-MS/MS analyses were carried out with an Shimadzu Prominence ultrafast liquid chromatography (UFLC) connected with an Applied Biosystem 5500 Q-Trap mass detector equipped with electrospray ionization source (ESI). Samples were separated on a Phenomenex C18 column (50 × 2.0 mm, 5 μm) kept at 35 °C. The mobile phases consisted of 0.05% formic acid in water (solvent A) and acetonitrile (solvent B), and the flow was set at 0.4 mL min^−1^. The linear gradient elution program was as follows: 20% B (0–1 min), 20–80% B (1–2 min), 80–20% B (2–3 min), and 20% B (3–5 min). The parameters of mass spectrometer with ESI under positive electrospray ionization mode were set as follows: source temperature, 550 °C; the needle voltage, 4.5 kV; the curtain gas, the ion source gas 1 and the ion source gas 2 were set at 50 psi, 50 psi and 10 psi. MS data were acquired by multiple reaction monitoring (MRM) with *m*/*z* 154.2 → 137.1 (DA), *m*/*z* 184.4 → 166.2 (E), *m*/*z* 170.3 → 152.2 (NE), *m*/*z* 177.2 → 160.0 (5-HT), *m*/*z* 146.1 → 87.1 (Ach), *m*/*z* 140.1 → 123.1 (DHBA, IS).

#### 2.8.2. Determination of CORT and ACTH in serum

Serum samples were applied to measure the levels of CORT and ACTH by means of ELISA according to the manufacture's recommendations (Nanjing Jiancheng Bioengineering Institute, Inc., Nanjing, China). A linear regression equation of standard curve was developed based on the concentrations of standard substance and the OD value measured at 450 nm. Final absorbance values of samples were proportional to their levels which were expressed as ng mL^−1^ of starting plasma.

#### 2.8.3. Determination of T-AOC, SOD activities and MDA level in the frontal cortex

For the determination of T-AOC, SOD activities and MDA level, frontal cortex homogenate was prepared and centrifuged, and all procedures complied with the manufacturer's instructions in assay kits (Nanjing Jiancheng Bioengineering Institute, Inc., Nanjing, China). Briefly, T-AOC was detected by T-AOC Assay Kits with ferric reducing antioxidant power (FRAP) method and was expressed as mmol g^−1^ protein. SOD activity was assessed by SOD Assay Kits with nitroblue tetrazolium (NBT) method and was expressed as units per mg protein. The content of MDA equivalents was determined by thiobarbituric acid (TBA) method and expressed as units per mg protein.

### 2.9. Statistical analysis

Data were analyzed by SPSS statistical software (SPSS 19.0 Inc., Chicago, IL, USA). A one-way analysis of variance (One-way ANOVA) with least-significant difference (LCSI) test was used for inter-group comparison. The *p* value less than 0.05 was considered statistically significant. The results were expressed as mean ± standard error of mean (SEM).

## 3. Results

### 3.1. Identification of phenolic compounds from OSE by UPLC-DAD/Q-TOF MS analysis

After investigating the influence of mobile phase, kind of chromatographic column, column temperature, flow rate and the intra- and inter-day precisions of UPLC method, typical UPLC-MS profiles in the negative-ion mode of OSE was shown in ESI Fig. 2.† It could be seen that 17 peaks were detected, which were identified by matching with the molecular weight, MS/MS data, UV-visible spectral characteristics, and retention time. The information of 17 tentative identification were shown in Table 1.

**Table 1:** Identified constituents from okra seed extract based on UPLC-DAD/Q-TOF MS analysis

No.	*t*_r_ (min)	MS^−^ (*m*/*z*)	MS/MS (*m*/*z*)	Formula	Identification
1	5.07	1218.2329	913.1798	C_60_H_50_O_24_	Epigallocat tetramer
			609.1238		
			305.0684		
2	7.71	913.1882	305.0663	C_45_H_38_O_18_	Epigallocat trimmer
			609.1227		
3	14.30	289.0682		C_15_H_14_O_6_	Epicatechin
4	14.50	305.0654		C_15_H_14_O_7_	Gallocatechin
5	18.11	289.0735		C_15_H_14_O_6_	Catechin (std^a^)
6	18.85	477.1194	387.1658	C_22_H_22_O_12_	Isorhamnetin-3-*O*-β-d-glucoside
			299.0641		
			163.0414		
7	20.38	595.4586	300.0255	C_26_H_28_O_16_	Quercetin-3-*O*-β-d-xyl(1→2)-β-d-glucoside (std^a^)
8	21.98	609.4293	300.0311	C_27_H_30_O_16_	Quercetin-7-*O*-rutinoside
9	22.42	479.0829	317.0225	C_21_H_2_0O_13_	Myricetin-3-*O*-β-d-glucoside
			163.0011		
10	22.66	595.1301	300.0277	C_26_H_28_O_16_	Quercetin-3-*O*-β-d-xyl(1→2)-β-d-galactoside
11	23.14	625.1394	300.027	C_27_H_30_O_17_	Quercetin-3-*O*-gentiobioside (std^a^)
12	24.48	463.0878	300.026	C_21_H_20_O_12_	Isoquercitrin (std^a^)
13	24.67	609.1459	463.0863	C_27_H_30_O_16_	Rutin (std^a^)
			301.0346		
14	25.32	505.1017	446.0776	C_23_H_22_O_13_	Quercetin-3-*O*-β-d-6′′-(acetyl)glucopyranoside
			301.0360		
15	28.86	301.0363		C_15_H_10_O_7_	Quercetin (std^a^)
16	31.30	299.0226	285.0389	C_16_H_12_O_6_	Kaempferide (std^a^)
17	31.94	315.0535		C_16_H_12_O_7_	Isorhamnetin (std^a^)

a Identification by comparing with reference substances.

#### 3.1.1. Phenolic acids

According to previous literature, phenolic acids in okra seeds are mainly catechin derivatives.^16^ Considering catechins have characteristic UV/Vis spectra with maximum absorbance at 270 nm and no absorption at 330 or 350 nm, it could be concluded that peak 1–5 were catechin derivates. What's more, ESIMS spectra showed that molecular ion [M − H]^−^ at *m*/*z* 1218.2329 and 913.1798, and the fragment ion at *m*/*z* 305.0684 were existed in both spectra. Therefore, it could be concluded that the peak 1 and 2 were the epigallocat tetramer and epigallocat trimmer. The molecular ion [M − H]^−^ of peak 4 was at *m*/*z* 305.0652 which could be identified as epigallocat. As for the peak 5, MS/MS spectra showed that molecular ion [M − H]^−^ were at *m*/*z* 289.0735 and the retention time was same with catechin standard, which could prove peak 5 was catechin. Based on the MS/MS spectra, the peak 3 was tentatively assigned as epicatechin.

#### 3.1.2. Flavonols

The second group of compounds included peaks 6–17, and these compounds were generally eluted later than the phenolic acids and displayed UV/Vis spectra with two absorption maxima at 240–280 nm (Band II) and 300–380 nm (Band I). As for the identification of these peaks, firstly, peak 7, 11, 12, 13, 15, 16, 17, identified by comparing the retention time and MS/MS spectra with available standards, were quercetin-3-*O*-β-d-xyl(1→2)-β-d-glucoside, quercetin-3-*O*-gentiobioside, isoquercitrin, rutin, quercetin, kaempferide, isorhamnetin. The molecular ion of peak 6 was at *m*/*z* 477.1194, and the main fragment ion was at 299.0614. From these, it could be detected that peak 6 was isorhamnetin-3-*O*-glucoside. The ESIMS spectra of peak 8, 10, 15 showed that their main fragment ions were at 301.03, which could prove that all these peaks were quercetin derivatives. Considering their molecular ions and other fragment ions, it could be concluded that peak 8, 10, 14 were quercetin-7-*O*-rutinoside, quercetin-3-*O*-β-d-xyl(1→2)-β-d-galactoside and quercetin-3-*O*-β-d-6′′-(acetyl)glucopyranoside. The molecular ion of peak 9 was at *m*/*z* 479.0829, and the main fragment ions were at *m*/*z* 317.0225 and 163.0011, which proved that the peak 9 was myricetin-3-*O*-β-d-glucoside.

In conclusion, as shown in the Table 1, 17 compounds, including 5 phenolic acids and 12 flavonoids, were identified or tentatively characterized by analyzing the MS/MS data and comparing with literature data and reference substances.

### 3.2. Antidepressant effects of OSE on sub-chronic treatment in mice

#### 3.2.1. Open field test

**Fig. 2:**
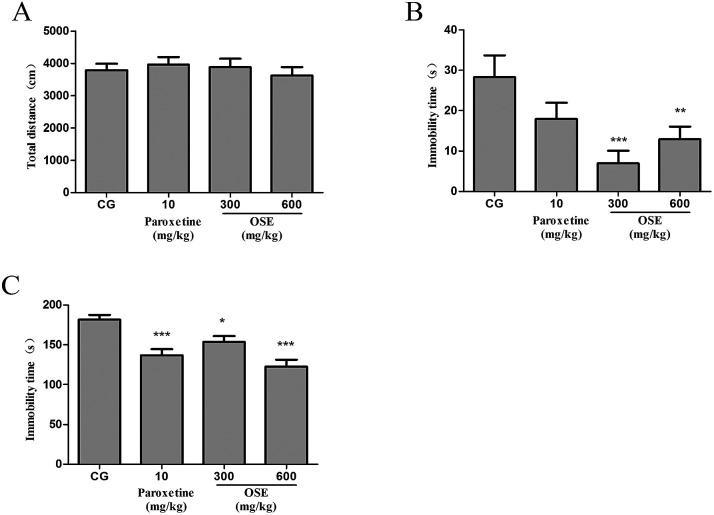
Effect of okra seed extract (OSE) on locomotive activities in the open field test (A), immobility time in tail suspension test (B) and immobility time in forced swim test (C) for sub-chronic treatment mice. *p < 0.05, **p < 0.01, ***p < 0.001 compared with control group (CG).

As shown in Fig. 2(A), no significant effect of OSE (300 and 600 mg kg^−1^) on the total distance of mice was observed in the open-field test, indicating OSE could not affect the mice locomotive activities.

#### 3.2.2. Tail suspension test

As revealed in Fig. 2(B), OSE treatment for 9 days at doses of 300 and 600 mg kg^−1^ significantly reduced immobility time as compared with control mice (*p* < 0.01 and *p* < 0.05). The positive control (paroxetine, 10 mg kg^−1^) showed a trend to reduce immobility time as compared with control mice.

#### 3.2.3. Forced swim test

As shown in Fig. 2(C), paroxetine (10 mg kg^−1^) and OSE (300 and 600 mg kg^−1^) treatment for 10 days significantly reduced immobility time as compared with control mice (*p* < 0.001, *p* < 0.05 and *p* < 0.001, respectively).

### 3.3. Antidepressant effects of OSE on chronical sleep interruption (CSI) mice

#### 3.3.1. Novelty suppressed feeding test

**Fig. 3:**
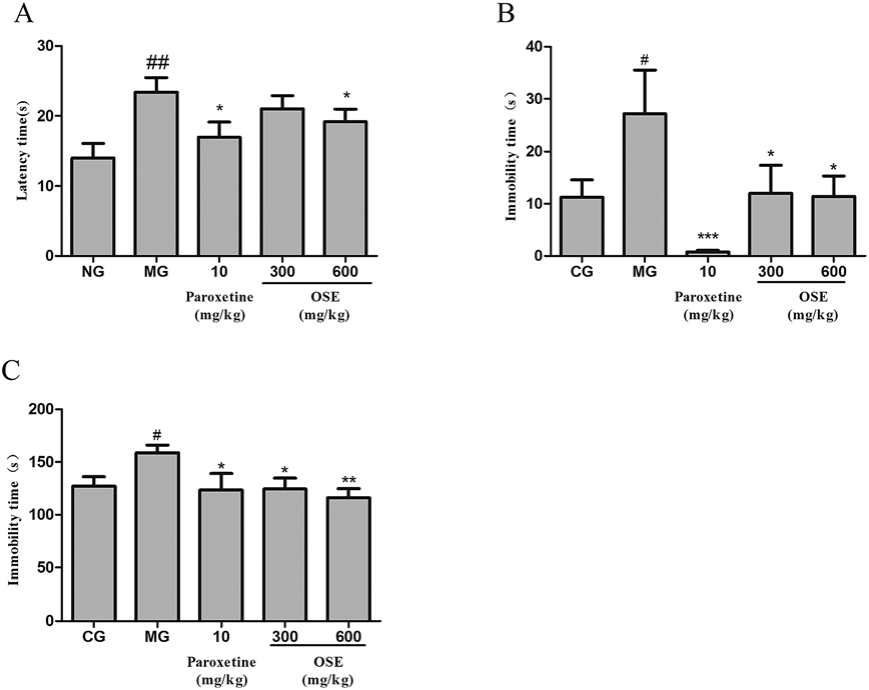
Effect of okra seed extract (OSE) on latency time in novelty suppressed feeding test (A), immobility time in tail suspension test (B) and immobility time in forced swim test (C) for chronical sleep interruption (CSI) mice. ^#^*p* < 0.05, ^##^*p* < 0.01 compared with control group (CG).*p < 0.05, **p < 0.01 compared with model group (MG).

As shown in the Fig. 3(A), the mice of model group had a significantly longer latency time to bite the chow than the control mice (*p* < 0.01). On the other hand, compared to the model group, this increased latency time was significantly attenuated by paroxetine (*p* < 0.05) and high dose (600 mg kg^−1^) of OSE (*p* < 0.05). The low dose (300 mg kg^−1^) of OSE had a trend to decrease the latency time of mice compared to the model group, but there is no significant difference between them.

#### 3.3.2. Tail suspension test

As expected from the Fig. 3(B), it could be seen that the immobility time of model group was significantly longer than that of normal group (*p* < 0.05). When compared with the model group, mice treated with paroxetine (*p* < 0.001) and OSE (*p* < 0.01 for low and high doses) showed shorter immobility time, which proved that depressive-like behaviour was prevented by the administration of paroxetine and OSE.

#### 3.3.3. Forced swimming test

As shown in the Fig. 3(C), the immobility time of model group was significantly increased compared to the normal group (*p* < 0.05). As for treatment groups, paroxetine (*p* < 0.05), low dose (300 mg kg^−1^) and high dose (600 mg kg^−1^) of OSE (*p* < 0.05 and *p* < 0.01) could significantly decrease the immobility time of mice as compared with model group.

#### 3.3.4. Results of neurotransmitter determination

**Fig. 4:**
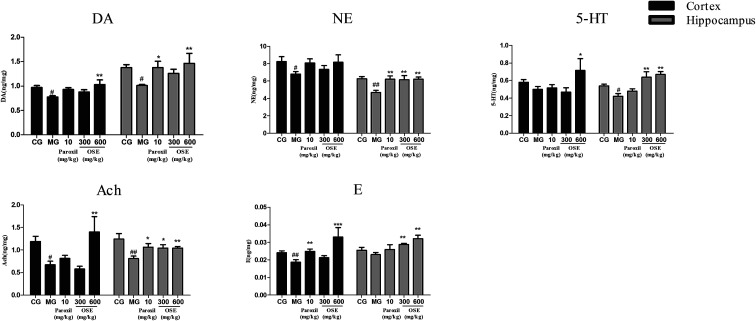
Effect of okra seed extract (OSE) on neurotransmitters in the hippocampus and frontal cortex of chronical sleep interruption (CSI) mice. Data are expressed as mean ± SEM. (*n* = 10). ^#^*p* < 0.05, ^##^*p* < 0.01 compared with control group (CG). *p < 0.05, **p < 0.01, ***p < 0.001 compared with model group (MG). DA: dopamine; NE: noradrenaline; 5-HT: 5-hydroxytryptamine; Ach: acetylcholine; E: epinephrine.

The contents of neurotransmitters in the hippocampus and frontal cortex after 21 days treatment are shown in Fig. 4. After 3 weeks of CSI procedure, the contents of DA, Ach and NE in model groups were significantly lower than those in control group in both hippocampus and frontal cortex, and the contents of 5-HT in model group were significantly lower than those in control group in hippocampus. Apart from these, for the model group, the content of 5-HT in cortex and the contents of E in both hippocampus and frontal cortex showed the decreased trends but not significant compared to the control group. Compared to the model group, chronic treatment with paroxetine (10 mg kg^−1^) and OSE (300, 600 mg kg^−1^) could increase all these neurotransmitter levels in the hippocampus, and the DA, NE, 5-HT, Ach and E levels in high dose of OSE group all showed the significantly increase. As for the frontal cortex, except the NE, the contents of other neurotransmitters in high dose of OSE group showed the significant increase compared with the model group.

#### 3.3.5. Results of CORT and ACTH determination

**Fig. 5:**
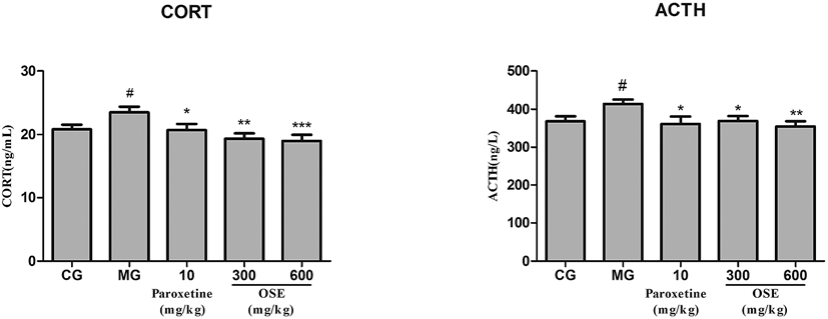
Analysis of CORT, ACTH in serum for chronical-sleep interruption (CSI) mice. Data are expressed as mean ± SEM. (*n* = 10). ^#^*p* < 0.05 compared with control group (CG). *p < 0.05, **p < 0.01, ***p < 0.001, compared with model group (MG). CORT: corticosterone; ACTH: adrenocorticotrophic hormone.

From the Fig. 5, it could be seen that CSI could significantly improve the contents of CORT and ACTH compared to the control group (both *p* < 0.05). After treatment of paroxetine and OSE (300, 600 mg kg^−1^), the contents of CORT and ACTH were markedly decreased (*p* < 0.05 or *p* < 0.001).

#### 3.3.6. Results of T-AOC, SOD activities and MDA level determination in frontal cortex

**Fig. 6:**
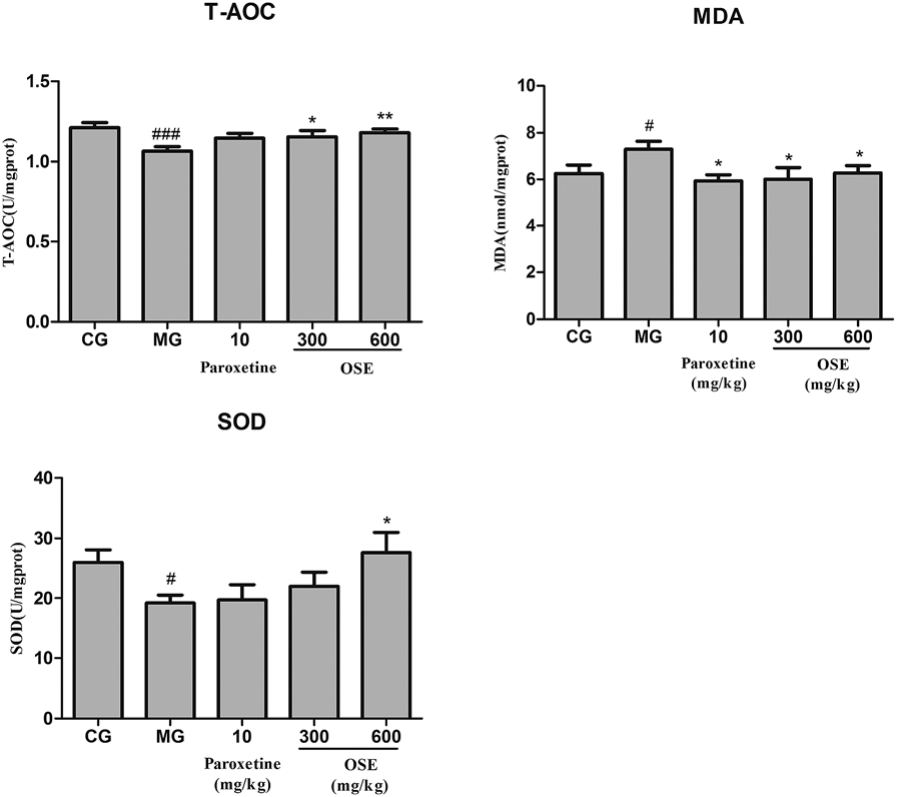
Analysis of T-AOC, SOD activity and MDA in frontal cortex for chronical-sleep interruption (CSI) mice. Data are expressed as mean ± SEM. (*n* = 10). ^#^*p* < 0.05 compared with control group (CG). *p < 0.05, **p < 0.01, ***p < 0.001, compared with model group (MG). T-AOC: total antioxidant capacity; SOD: superoxide dismutase; MDA: malonaldehyde.

In Fig. 6, it could be seen that CSI could significantly decrease activities of T-AOC and SOD, and improve the content of MDA in model group compared to control group (*p* < 0.001; *p* < 0.05 and *p* < 0.05, respectively). And the treatment of OSE (300, 600 mg kg^−1^) could significantly promote the contents of T-AOC in both groups (*p* < 0.05 and *p* < 0.01) and enhance the activity of SOD in high-dose OSE group (*p* < 0.05). Besides, the paroxetine group (10 mg kg^−1^) and OSE groups (300, 600 mg kg^−1^) could significantly decrease MDA level (*p* < 0.05 for three groups).

## 4. Discussion

To the best of our current knowledge, it is the first time to demonstrate the antidepressant effects of OSE using both sub-chronic and CSI animal models. From the results of present research, it could be concluded that supplement of OSE could inhibit depression-like behavior by suppressing the hyperactivation of hypothalamic–pituitary–adrenal (HPA) axis, alleviating oxidative stress and normalizing the levels of neurotransmitters in the hippocampus and frontal cortex.

TST and FST are two validated models used to assess putative antidepressant compounds, which are based on the fact that animals subjected to short-term, inescapable stress can develop an immobile posture. The immobility, referred to as behavioral despair in animals, is claimed to reproduce a condition similar to human depression.^26^ In the present study, oral treatment with OSE for 10 days significantly reduced the immobility time in TST and FST, suggesting that OSE has antidepressant effects on mice. To exclude the possibility that the antidepressant-like effects of OSE were attributable to stimulatory effects on locomotor function, OSE-induced spontaneous activity was evaluated in the open field test. Results revealed OSE-treated mice exhibited no alterations in spontaneous activity, indicating that the decreases in immobility time in the FST and TST were not caused by motor stimulation but rather to increase in active movements, such as struggling and swimming.

Based on the sub-chronic experiment, the effect of OSE on CSI mice was evaluated. CSI model can induce depression-like behavioral alterations, and is usually used for evaluating the efficacy of antidepressants through behavioral tests, such as FST, TST and NSFT. Both FST and TST are mentioned above. The NSFT is usually employed to define anhedonia (a loss of responsiveness to pleasant events), which is a core symptom in the diagnosis of depression and can be modeled by inducing a reduction of sucrose consumption in depression-like animal models.^27^ In the present study, as can be seen from Fig. 3, CSI induced significant increase of immobility in the FST and TST, and the latency in NSFT compared with control group. However, treatment with OSE (300 and 600 mg kg^−1^) and paroxetine (10 mg kg^−1^) were found to prevent changes induced by CSI. These results demonstrated OSE possessed antidepressant effects in the CSI model.

Currently, the pathogenesis of depression remains poorly understood. Among several putative hypothesis, stress is one of the most important factors responsible for depressive disorders, and the hyperactivation of hypothalamic–pituitary–adrenal (HPA) axis has attracted much more appreciation. Corticotrophin releasing factor (CRF) is produced from the paraventricular nucleus (PVN) to induce adrenocorticotropic hormone (ACTH) release from the pituitary, which in turn stimulates glucocorticoids (GCs) from the adrenal.^28^ Under normal conditions, glucocorticoid level in blood is sensitively regulated by HPA axis *via* negative feedback; when chronically stressful situation, such as CSI or chronic unpredictable mild stress (CUMS) models, occurs, HPA axis will be activated *via* stimulation of adrenocorticotropic hormone (ACTH) release and the subsequent peripheral release of steroids/cortisol from the adrenal grand. The persistently high concentration of blood glucocorticoids causes the dysfunction of HPA axis, exacerbates the lesion in the nervous system, and even aggravates the depression.^29,30^ In present research, it was observed that CSI mice displayed an obvious elevation of GC and ACTH levels in serum. However, OSE treatment could significantly reversed the elevated trend of GC and ACTH levels in CSI mice, indicating the alleviation of depression severity.

Growing evidence suggests that oxidative stress plays an important role in the development of chronic diseases such as cardiovascular disease and some psychiatric disorders such as depression. The brain is particularly vulnerable to oxidative stress due to a high consumption of oxygen, the generation of reactive oxygen species (ROS), high long chain polyunsaturated fatty acid content (which, in turn, are vulnerable to ROS attacks) and relatively poor antioxidant capacity. When living cells are threatened by certain physical and psychological events, it will lead to oxidative damage. The oxidative damage could be indicated by level of MDA, a product of lipid peroxidation, and the activity of antioxidant enzymes such as SOD and T-AOC. In present study, administration of OSE reversed the increase of MDA in the hippocampus of mice induced by CSI significantly, while obviously increased the activities of SOD and T-AOC as compared with the model mice. The data suggested the antioxidant activity of OSE have a potential relationship with its antidepressant effects.

It is well known that currently treatment of MDD is focused on the monoamine hypothesis, which prop that depression is related to the low-level of brain monoamines including serotonin (5-HT), dopamine (DA) and norepinephrine (NE).^31^ 5-HT is involved in many neurological and psychiatric diseases related to mood, sleep, learning, memory and sexual behavior;^32^ NE has long been known to play prominent roles in aggression, anxiety, reward and other stress-related behaviors;^33^ dopaminergic pathways are more likely altered in psychomotor retarded depressive patients.^34^ Additionally, increasing number of evidences indicate that MDD is associated with altered function of the major excitatory and inhibitory neurotransmitters. Peripheral administration of the acetylcholinesterase (AChE) antagonist physostigmine induces symptoms of anxiety and depression in human subjects by decreasing the breakdown of Ach and increasing level of Ach in the brain.^35^ To detect the brain monoamine and Ach levels in CSI mice, the hippocampus and the frontal cortex, which are critically involved in the regulation of emotion, motivation, learning and memory, were investigated in present study. Our results revealed that OSE (600 mg kg^−1^) led to a marked increase of DA, NE, 5-HT, Ach and E levels in hippocampus, and also promoted the levels of DA, 5-HT, Ach and E levels in frontal cortex. These results demonstrated that OSE could promote levels of hippocampus and frontal cortex monoamines to display antidepressant activity.

When it comes to the chemical compositions of OSE, various chromatographic methods were used to isolate 14 compounds from OSE, and their structures were identified based on spectrum data of ^1^H NMR, ^13^C NMR and MS as shown in ESI Fig. 1.† Based on these isolated substances, the chemical compositions of OSE were further analyzed with UPLC-DAD/Q-TOF MS. UPLC is a novel approach to chromatographic separation, which is based on the use of columns with smaller packing (1.7 μm particle) and operated at higher pressures (up to 15 000 psi). Compared with traditional HPLC, UPLC provides a higher peak capacity, greater resolution, increased sensitivity and higher speed of analysis. When UPLC is coupled to mass spectrometry, it has been widely used as a powerful tool in the analysis of characterization and quantification of different complicated samples. In present study, 17 phenolic compounds, including epigallocat tetramer, epigallocat trimmer, epicatechin, gallocatechin, catechin, isorhamnetin-3-*O*-β-d-glucoside, quercetin-3-*O*-β-d-xyl(1→2)-β-d-glucoside, isoquercitrin, rutin, quercetin-7-*O*-rutinoside, myricetin-3-*O*-β-d-glucoside, quercetin-3-*O*-β-d-xyl(1→2)-β-d-galactoside, quercetin-3-*O*-gentiobioside, quercetin-3-*O*-β-d-6′′-(acetyl)glucopyranoside, quercetin, kaempferide and isorhamnetin from okra seed extract were identified using UPLC-DAD/ESI-Q-TOF MS. Our results revealed that OSE mainly contained catechin and quercetin derivatives, which is consistant with the previous report.^16^ As for the relationship between the active ingredients and curative effects of OSE, it can be concluded that phenolic compounds play the important roles for its antidepressant activity. Numerous previous literatures have proved that phenolic acids and quercetin-related flavonoids showed antidepressant activity due to their antioxidant effects.^36,37^ Meanwhile, the results of chemical analysis in present study showed that the catechin and quercetin derivatives are the major compositions of OSE.

## 5. Conclusions

The present study firstly demonstrated OSE possessed antidepressant effects on sub-chronic treatment mice and CSI animal model through behavior tests, which. And the potential mechanisms may be *via* dampening oxidative stress, supressing HPA axis, as well as regulating the neurotransmitter levels. UPLC-DAD/Q-TOF MS analysis showed OSE mainly contained catechin and quercetin derivative. From the present data, the phenolic compounds should be the antidepressant constituents of OSE.

## Conflicts of interest

There are no conflicts to declare.

## Supplementary Material

RA-008-C8RA03201G-s001
