# Supplementary material for: Antidepressant activity of an aqueous extract from okra seeds
Source: RSC Adv. 2018 Sep 21;8(57):32814–22. doi: 10.1039/c8ra03201g (PMC9086372; doi:10.1039/c8ra03201g)
Supplement: RA-008-C8RA03201G-s001 [file RA-008-C8RA03201G-s001.pdf]

Electronic Supplementary Material (ESI) for RSC Advances.
This journal is © The Royal Society of Chemistry 2018

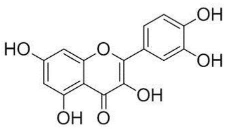

Quercetin

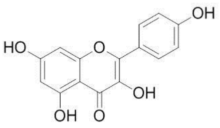

Kaempferide

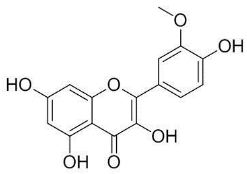

Isorhamnetin

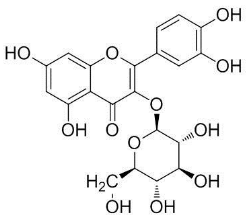

Isoquercitrin

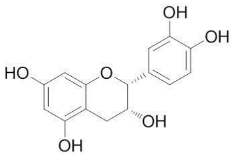

Epicatechin

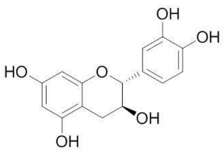

Catechin

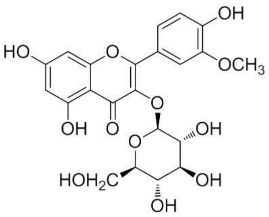

Isorhamnetin-3-O-β-D-glucoside

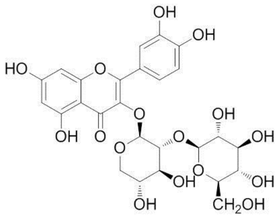

Quercetin-3-O-β-D-xyl(1→2)-β-D-glucoside

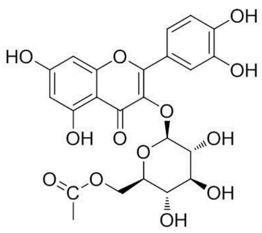

Quercetin-3-O-β-D-6"-(acetyl) glucopyranoside

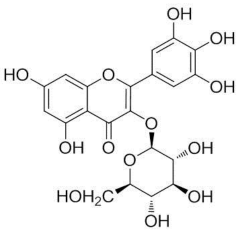

Myricetin-3-O-β-D-glucoside

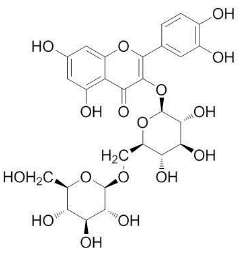

Quercetin-3-O-gentiobioside

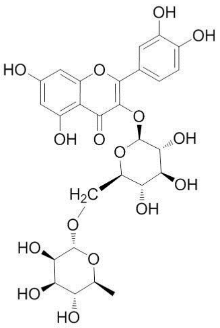

Rutin

Supplementary Figure 1. Structure of fourteen reference compounds

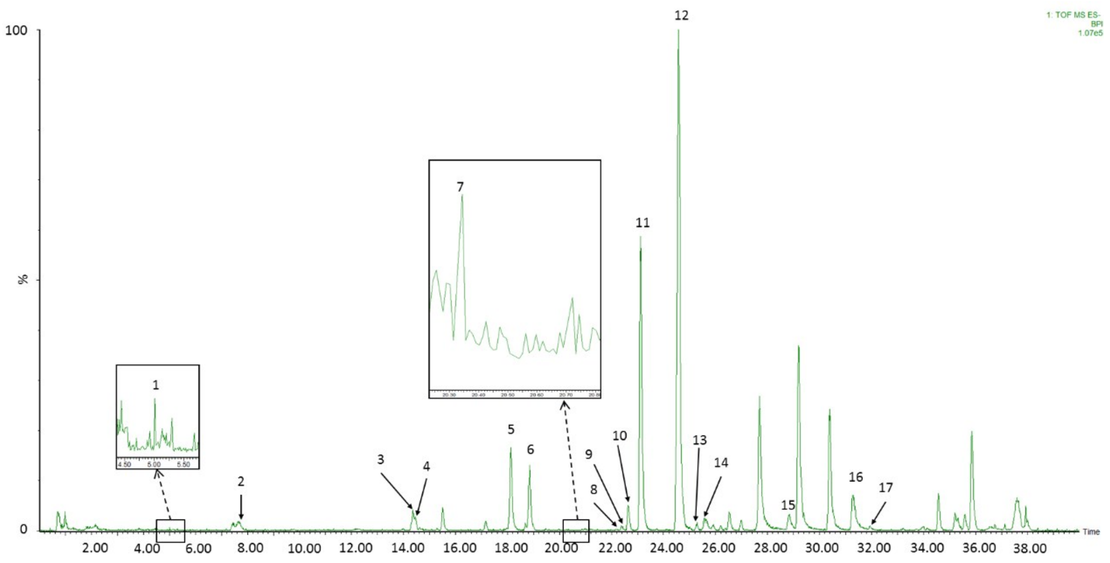

Supplementary Figure 2. Total Ion Chromatogram of Okra Seed extract (OSE) under the Negative Ion Mode
